# Prognostic value of elevated plasma angiotensin-converting enzyme 2 in cardiometabolic diseases: A review

**DOI:** 10.1097/MD.0000000000033251

**Published:** 2023-03-10

**Authors:** Gang Zhou, Jingchen Liu

**Affiliations:** a Department of First Clinical Medical College, Guangxi Medical University, Nanning, Guangxi, China; b Department of Anesthesiology, the First Affiliated Hospital of Guangxi Medical University, Nanning, Guangxi, China.

**Keywords:** angiotensin-converting enzyme 2, biomarker, cardiometabolic disease, chronic kidney disease, COVID-19, diabetes

## Abstract

Angiotensin-converting enzyme 2, as an internal anti regulator of the renin-angiotensin hormone cascade reaction, plays a protective role in vasodilation, inhibition of fibrosis, and initiation of anti-inflammatory and antioxidative stress by degrading angiotensin II and generating angiotensin (1–7). Multiple studies have shown that plasma angiotensin-converting enzyme 2 activity is low in healthy populations without significant cardiometabolic disease, and elevated plasma angiotensin-converting enzyme 2 levels can be used as a novel biomarker of abnormal myocardial structure and/or adverse events in cardiometabolic diseases. This article aims to elaborate the determinants of plasma angiotensin-converting enzyme 2 concentration, the relevance between angiotensin-converting enzyme 2 and cardiometabolic disease risk markers, and its relative importance compared with known cardiovascular disease risk factors. Confronted with the known cardiovascular risk factors, plasma angiotensin-converting enzyme 2 (ACE2) concentration uniformly emerged as a firm predictor of abnormal myocardial structure and/or adverse events in cardiometabolic diseases and may improve the risk prediction of cardiometabolic diseases when combined with other conventional risk factors. Cardiovascular disease is the leading cause of death worldwide, while the renin-angiotensin system is the main hormone cascade system involved in the pathophysiology of cardiovascular disease. A multi-ancestry global cohort study from the general population by Narula et al revealed that plasma ACE2 concentration was strongly associated with cardiometabolic disease and might be an easily measurable indicator of renin-angiotensin system disorder. The association between this atypical hormone disorder marker and cardiometabolic disease is isolated from conventional cardiac risk factors and brain natriuretic peptide, suggesting that a clearer comprehending of the changes in plasma ACE2 concentration and activity may help us to improve the risk prediction of cardiometabolic disease, guide early diagnosis and feasible therapies, and develop and test new therapeutic targets.

## 1. Introduction

Cardiovascular disease is the leading cause of death worldwide,^[1]^ while the renin-angiotensin system is the main hormone cascade system involved in the pathophysiology of cardiovascular disease. A multi-ancestry global cohort study from the general population by Narula et al^[2]^ revealed that plasma ACE2 concentration was strongly associated with cardiometabolic disease and might be an easily measurable indicator of renin-angiotensin system disorder. The association between this atypical hormone disorder marker and cardiometabolic disease is isolated from conventional cardiac risk factors and brain natriuretic peptide, suggesting that a clearer comprehending of the changes in plasma ACE2 concentration and activity may help us to improve the risk prediction of cardiometabolic disease, guide early diagnosis and feasible therapies, and develop and test new therapeutic targets.

In the renin-angiotensin system (RAS), the angiotensin-converting enzyme (ACE) converts angiotensin (Ang) I into angiotensin II (Ang II) which mediates vasoconstriction, sodium and water retention, cardiac remodeling, fibrosis, inflammation, endothelial dysfunction, and oxidative stress through the angiotensin type 1 receptor.^[3]^ As an endogenous anti-regulatory factor for the renin-angiotensin hormone cascade reaction, ACE2 converts Ang I and Ang II (the preferred substrate, the affinity of ACE2 to Ang II is about 400 times higher than that of Ang I.^[4]^ Therefore, ACE2 mainly hydrolyzes Ang II in vivo) into Ang (1–9) and Ang (1–7).^[5]^ Ang (1–9) has a biological effect on anti-myocardial fibrosis through the Ang II type 2 receptor.^[6,7]^ Ang (1–7) can also be generated by ACE cleavage of Ang (1–9), which acts on the G protein-coupled receptor Mas pathway,^[8,9]^ and plays a protective role in vasodilation, inhibition of fibrosis, and initiation of anti-inflammatory and antioxidant stress.^[10]^ ACE2 counteracts the effect of Ang II by degrading Ang II and generating Ang (1–7). The ultimate effect of RAS activation depends on the balance between plasma Ang (1–7)/ Ang II levels, which determines the availability of different angiotensin peptides and thus determines the balance between pro-inflammatory and anti-inflammatory pathways as well as pro-fibrotic and antifibrotic pathways. Decreased Ang (1–7)/ Ang II ratio was related to worsening cardiovascular adverse events and longer hospitalization.

## 2. Study of plasma ACE2

### 2.1. Biochemistry and regulation of ACE2

ACE2 is the first homologous gene of human ACE, cloned from the cDNA library of human lymphoma and heart failure patients.^[11]^ ACE2 is an intact type 1 membrane protein that is highly expressed in the heart, blood vessels, lungs, and kidneys.^[5,12,13]^ It exists not only in membrane-bound cells but also in free form in circulating plasma by the cleavage of the extracellular domain protein by (tumor necrosis factor-α converting enzyme also known as ADAM17, a resolvase, and metalloproteinase).^[14]^ Studies have shown that there were 2 different soluble forms of ACE2 shedding in primary cultured human airway epithelial cells.^[15]^ This indicates that the shedding process may involve 1 or more enzymes, and further research is required to determine the mechanism of ACE2 shedding as well as the involvement of other shedding enzymes and their real cleavage sites. The mechanism of elevated plasma ACE2 concentration is still an active research field, which may be a complex interaction involving cell expression, hydrolysis of shedding enzymes, and impaired plasma clearance rate which affects plasma concentration. Previous studies have revealed that ACE2 might be posttranscriptional regulated by miR-421 which can be used as a novel potential therapeutic target to modulate ACE2 expression in diseases.^[16]^

### 2.2. Genotyping and genetic analysis

Narula et al^[2]^ found that 2 gene loci associated with ACE2 concentration had genome-wide significance through genome-wide meta-analysis. One of the gene loci was the variant rs5936022 encoded by the X chromosome. The increased variant rs5936022 was linked with the elevated ACE2 expression in multiple brain regions.^[17,18]^ Another rs2464190, located upstream of the HNF1A gene locus, might be the main regulator of ACE2 levels since its promoter region possesses 3 HNF1A combining sites. HNF1A could induce upper cellular ACE2 expression in islet cells,^[19]^ this special variation was also associated with the susceptibility of coronary artery disease and type 2 diabetes.^[20,21]^

### 2.3. The relevance between ACE2 and cardiometabolic disease risk factors

Multiple studies have revealed that plasma ACE2 activity was low in healthy population without significant cardiometabolic disease, and elevated plasma ACE2 levels could be used as a novel predictor of abnormal myocardial structure and/or adverse events in cardiometabolic diseases involving heart failure (HF), myocardial infarction, atrial fibrillation, coronary artery disease, stroke, and aortic stenosis.^[22–28]^ Elevated plasma ACE2 activity also was an independent predictor of sensitivity and specificity for all-cause mortality.^[23,28]^ Compared with the established clinical risk factors (smoking, diabetes, hypertension, hyperlipidemia, and high body mass index), plasma ACE2 concentration consistently emerged as a strong predictor of cardiovascular diseases or death.^[2]^

### 2.4. The activity of ACE2

Plasma ACE2 activity was inhibited by endogenous inhibitor in vivo, this inhibition was dose-dependent. the endogenous inhibitor could be removed by anion exchange chromatography,^[29]^ and was not affected by decreased renal function,^[30]^ but it is still unclear whether other cardiovascular risk factors or diseases influence internal inhibitor levels. The study has shown that plasma ACE2 levels might be parallel to the expression of tissue ACE2 and the constant shedding rate in normal physiology.^[31]^ In the study by Narula et al^[2]^, the gene variation rs5936022, associated with plasma ACE2 levels, was related to elevated ACE2 expression in the vascular system, brain as well as heart, indicating that elevated plasma ACE2 levels reflected elevated ACE2 synthesis in tissue. On the contrary, Ramchand et al^[28]^ have shown that elevated plasma ACE2 concentration was associated with decreased myocardial ACE2 gene expression in patients with aortic stenosis. It remains to be a problem that how to simultaneously measure the ACE2 activity in human tissues and plasma to identify whether increased plasma ACE2 activity is related to elevated ACE2 mRNA synthesis and/or elevated ACE2 shedding in tissues.

### 2.5. Determinants of plasma ACE2 concentration

Studies have shown that plasma ACE2 levels in males were significantly higher than in females.^[23,25]^ The difference in ACE2 expression between genders is also recorded in diverse human tissues involving the renal cortex, heart, and adipose tissue.^[18]^ Considering that the genes encoding ACE2 are located on the X chromosome, X chromosome deactivation and escapement may lead to phenotypic differences and tissue specificity differences between genders.^[32]^ Although men are associated with a higher concentration of ACE2, There is no significant evidence to suggest that ACE2 has a heterogeneous effect on major adverse cardiovascular outcomes between men and women.^[2]^ Poorly is known about the biological significance of elevated ACE2 concentration in men and its relationship with gender susceptibility to cardiovascular diseases. Narula et al^[2]^ revealed that gender was the most important factor determining the plasma ACE2 concentration, followed by race, body mass index, diabetes, age, systolic blood pressure, smoking status, and low-density lipoprotein. To investigate whether there is a potential causal relationship between clinical risk factors and ACE2 levels, Narula et al^[2]^ showed that genetically higher body mass index and higher risk of type 2 diabetes were associated with elevated plasma ACE2 levels through the Mendelian randomization analysis, However, there was no significant correlation between genetic susceptibility to smoking, elevated low-density lipoprotein and systolic blood pressure with plasma ACE2 levels. In regression analyses of patients with heart failure, ACE2 activity was not associated with glomerular filtration rate and C-reactive protein.^[22]^

### 2.6. ACE2 and antihypertensive therapies

ACE inhibitors do not seem to affect ACE2 activity because they act on different sites.^[33]^ Previous studies have also confirmed that there was no significant difference in plasma ACE2 levels between the use of angiotensin receptor blockers, ACE inhibitors, calcium channel blockers, β blockers, or diuretics.^[2,28]^

## 3. ACE2 and cardiovascular disease

Previous studies have evaluated the prognosis value of plasma ACE2 levels in patients with confirmed cardiovascular disease. In patients with obstructive coronary artery disease, HF, aortic stenosis as well as atrial fibrillation, elevated ACE2 activity was related to the elevated risk of cardiac dysfunction and adverse outcomes.^[23–25,28]^ This means that increased plasma ACE2 activity may act as a positively biological indicator of these diseases or their severity. Plasma ACE2 levels also reflect changes in cardiac structure. In patients with atrial fibrillation, elevated plasma ACE2 activity was related to left atrial structural remodeling.^[24]^ In patients with HF, increased plasma ACE2 levels independently predicted cardiovascular outcomes (hospitalization of heart failure, heart transplantation, or death) and were positively related to imaging indicators of ventricular remodeling as well as function disorder after 34 months of follow up.^[31]^ Similarly, plasma ACE2 activity was associated with infarct size and more severe left ventricular remodeling after myocardial infarction with ST-segment elevation.^[26]^

### 3.1. ACE2 and coronary artery disease

Previous studies have shown a significant increase in plasma ACE2 activity after myocardial infarction.^[26]^ However, Ramchand et al^[23]^ showed that plasma ACE2 activity increased even without acute myocardial injury in patients with obstructive coronary disease, which might reflect the potential atherosclerosis of the coronary artery rather than myocardial infarction. This suggests that plasma ACE2 activity may be an indicator of atherosclerosis. In patients with non-dialysis chronic kidney disease, circulating ACE2 activity was associated with silent atherosclerosis in carotid and peripheral vessels.^[34]^ Studies have shown that ACE2 was expressed in vascular endothelial cells, macrophages, and smooth muscle cells in aortic and coronary atherosclerotic lesions.^[35,36]^ ACE2 was present in atherosclerotic blood vessels during coronary artery bypass surgery in patients with coronary heart disease.^[10]^ Experimental studies have shown that ACE2 overexpression could promote the stability of atherosclerotic plaques and reduce atherosclerotic lesions.^[30]^ ACE2 tissue activity was lower in stable advanced atherosclerotic lesions than in early and ruptured atherosclerotic lesions.^[35]^ In the rabbit atherosclerosis model, silencing the tumor necrosis factor-α converting enzyme gene enhanced plaque stability and improved vascular remodeling.^[37]^ These findings reinforce the important anti-regulation role of ACE2 in atherosclerosis and suggest that the regulation of ACE2 may provide an option for the treatment of patients with atherosclerosis in the future.

### 3.2. ACE2 and heart failure

In patients with suspicious HF, serum ACE2 activity was firmly related to brain natriuretic peptide (BNP) levels, left ventricular ejection fraction, and clinical diagnosis of HF. Úri et al^[22]^ demonstrated that serum ACE2 activity had a diagnostic value in distinguishing heart failure with preserved ejection fraction (HFpEF) from heart failure with reduced ejection fraction (HFrEF) and it was associated with cardiovascular disease progression, ACE2 activity was significantly increased in hypertensive state and further increased in HFrEF but not in HFpEF when hypertension develops into HF. This indicates that serum ACE2 activity may be a selective biomarker for systolic dysfunction and has no correlation with the severity of diastolic dysfunction. Compared with serum ACE2 activity, plasma BNP and amino-terminal pro-B-type natriuretic peptide levels are forceful independent predictors of heart failure incidence and mortality, but they have poor selectivity to distinguish HFpEF from HFrEF because they have barely statistical difference.^[38]^ Current studies of heart failure showed a significant positive relevance between circulating ACE2 activity and BNP levels.^[23,28]^ In Chagas disease, Plasma ACE2 activity was an independent prognostic biological marker and similarly valid to BNP, the combination of ACE2 activity and BNP levels enhanced the predictive value of adverse cardiac outcomes.^[39]^ Compared with patients without ACE2 or BNP elevation or only 1 biomarker elevation, patients with elevated plasma ACE2 activity and elevated BNP were more likely to have higher mortality. Since the determination of plasma ACE2 has a prognostic value that cannot be detected by BNP alone, the combination of these 2 biological markers may improve the medical decision-making capacity of aortic stenosis. Importantly, more than 40% of patients with elevated ACE2 activity had normal BNP levels.^[28]^ Even if the extra adjustment for BNP, elevated ACE2 levels were still related to a higher risk of myocardial infarction, stroke, diabetes as well as death. The relationship between ACE2 and HF was corresponding but decreased after adjusting for BNP.^[2]^

### 3.3. ACE2 and aortic stenosis

In patients with aortic stenosis, increased plasma ACE2 concentration was related to decreased myocardial ACE2 gene expression, abnormal myocardial structure as well as more severe myocardial fibrosis, and was an independent predictor of mortality. Plasma ACE2 levels were correlated with the degree of valvular calcification and left ventricular end-diastolic volume, but not with left ventricular ejection fraction and the severity of aortic stenosis.^[28]^

### 3.4. ACE2 and stroke

The rat experiment showed that after 4 hours of focal ischemia caused by acute ischemic stroke, the serum ACE2 activity initially decreased, and then rebounded within 3 days.^[40]^ Preclinical studies on acute ischemic stroke indicated that the activation of ACE2/Ang (1–7)/Mas axis through targeted intervention could induce neuroprotective effects.^[41]^ Bennion et al^[27]^ showed that serum ACE2 activity was significantly correlated with ischemic stroke, but did not correlate with the infarction area of stroke. This study left 2 problems: using enzyme activity determination to provide data on the levels of functional plasma ACE2, but currently, there is no way to determine the tissue sources of ACE2. We can only speculate that the circulating ACE2 concentration is related to the tissue ACE2 concentration. ACE2 works in both membrane binding and soluble states. Ischemic cell death may lead to increased release of these 2 types, so it is not clear which 1 may be the main source of serum ACE2 activity during the stroke. Future studies need to assess the role and effect of ACE2 shedding in different tissues in stroke.

## 4. ACE2 and Ang (1–7)/Ang II ratio

The previous study has shown that the expression and shedding of ACE2 elevated under hypoxia.^[42]^ After the supplementation of recombinant Human ACE2(rhACE2), Ang II was effectively converted into Ang (1–7), significantly increasing the Ang (1–7)/Ang II ratio.^[43]^ Ang (1–7)/Ang II ratio undergoes dynamic changes in disease. In patients with chronic heart failure receiving angiotensin-converting enzyme inhibition, plasma Ang II was inhibited, while plasma Ang (1–7) was increased. On the contrary, plasma Ang II was increased and Ang (1–7) level was decreased in patients with acute heart failure receiving angiotensin receptor blockers.^[43]^ In the stage of chronic heart failure, elevated Ang (1–7) levels enhanced heart function and reversed pathological remodeling. When it reached the end-stage heart failure, tissue RAS was activated, which could result in increased myocardial Ang II levels and had nothing to do with systemic RAS. The circulating Ang II levels increased in patients above III degrees by the functional classification of the New York Heart Association (NYHA), while the circulating Ang (1–7) levels were higher in patients with NYHA functional class I/II degree. Regardless of ejection fraction, Ang (1–7)/Ang II ratio was significantly higher in patients with NYHA functional class I/II than III/IV.^[43]^ This suggested that ACE2 may act as a predictor for disease progression since it reflected the severity of HF in the NYHA functional classification. It also indicated that targeted therapies, gene therapy, for instance, aimed to improve Ang (1–7) levels, may be a feasible treatment strategy.

## 5. ACE2 and other diseases related to cardiovascular disorders

Roberts et al showed that the plasma ACE2 activity seemed to have increased in all chronic kidney disease (CKD) groups including predialysis, transplant patients, and even dialysis patients when compared with the historical samples of healthy subjects. this study suggested that although plasma ACE2 activity tends to increase in CKD patients, it may be a compensatory mechanism to reduce Ang II overactivity, and plasma ACE2 activity was relatively deficient at end-stage renal disease and dialysis initiation. This demonstrated that ACE2 may be involved as a novel biomarker in the progression of CKD.

Narula et al^[2]^ showed that elevated plasma ACE2 levels were related to the genetically upper body mass index and a biggish risk of type 2 diabetes. This manifested that ACE2 has a significant metabolic meaning. ACE2 provided significant renal protection in patients with diabetes, while ACE2 deficiency aggravated diabetic kidney injury, rhACE2 has therapeutic effects in experimental Alport syndrome and diabetic nephropathy.^[44,45]^ The results of Marfella et al^[46]^ showed that high glucose environment was more conducive to the formation of glycosylated ACE2, and the expression of glycosylated ACE2 in cardiomyocytes was strongly correlated with blood glucose control. ACE2 and glycosylated ACE2 were lower in patients with good glycemic control (HbA1c < 7%) than in patients with poor glycemic control (HbA1c > 7%). In other words, poor blood glucose control leads to increased glycosylated ACE2 levels in cardiomyocytes. Therefore, the expression of glycosylated ACE2 in type 2 diabetes mellitus (T2DM) patients is higher than that in non-T2DM patients, and promotes myocardial fibrosis by reducing the expression of Ang 1 to 9, Ang 1 to 7, and MasR, resulting in impaired cardiac function. Thus, the degree of myocardial fibrosis in T2DM patients is higher than that in non-T2DM patients.

Since the discovery of ACE2, more than 20 years of basic biological and physiological knowledge has been accumulated, many follow up studies have indicated that ACE2 may act as a potential target for the prevention and treatment of chronic inflammation and inflammatory diseases. Recently, ACE2 has received extensive attention as the cellular receptor of severe acute respiratory syndrome coronavirus 2 (SARS-CoV-2) which resulted in coronavirus disease (COVID-19)-related pneumonia. COVID-19 patients developed pneumonia due to accelerated injury in patients with multiple organ failures partly due to inflammatory cytokine storm induced by type 1 and type 2 T helper cells dysfunction and immune cells overactivation.^[47–51]^ Lung function and pathological damage were improved after injection of rhACE2, inflammation was also alleviated.^[52]^ Respiratory dysfunction and hypoxemia induced by patients with COVID-19, resulting in acute myocardial injury and chronic damage to the cardiovascular system. Severe symptoms are highly possible to occur in patients with cardiovascular disease if infected with SARS-CoV-2 which was triggered by the binding of the spike protein of the virus to ACE2. The binding decreased the protective function of ACE2. Interestingly, the trimer Spike protein in SARS-CoV-2-infected cells can be cleaved into *S*1 and *S*2 subunits, the *S*1 subunit contains a complete receptor-binding domain that binds to ACE2, thereby reducing the virus’s infectivity to neighboring cells by inducing ACE2 downregulation and reducing the number of ACE2 receptor molecules on its surface.^[53]^ D’Onofrio et al^[54]^ have shown that the expression of total ACE2 and glycosylated ACE2 in cardiomyocytes of DM patients is higher than that of non-DM patients, which is conducive to the entry of SARS-CoV-2 into host cells to increase the susceptibility of diabetic patients to COVID-19 infection and leads to worse prognosis. Therefore, reducing the expression of total ACE2 and glycosylated ACE2 may be a therapeutic target for DM patients to reduce COVID-19 infection.

## 6. Discussions

Numerous studies have shown that elevated plasma ACE2 levels could be used as a novel biological predictor of abnormal myocardial structure and/or adverse events in cardiometabolic diseases involving HF, myocardial infarction, aortic stenosis, atrial fibrillation, coronary artery disease, and stroke. The study of plasma ACE2 will help us to improve the risk prediction of cardiometabolic disease, guide clinically feasible therapies, and develop new therapeutic targets. However, future studies also need to solve the following problems: To determine the mechanism of ACE2 shedding, and the involvement of other shedding enzymes as well as the actual cleavage sites involved; How to simultaneously determine the ACE2 activity in tissues and circulation; The relationship between tissue ACE2 activity and circulating ACE2 activity; How to determine the critical value of plasma ACE2 to meet the optimal combination of sensitivity and specificity simultaneously.

## Author contributions

**Conceptualization:** Gang Zhou.

**Formal analysis:** Gang Zhou.

**Software:** Gang Zhou.

**Supervision:** Jingchen Liu.

**Writing – original draft:** Gang Zhou.

**Writing – review & editing:** Gang Zhou, Jingchen Liu.
